# Gender differences in right ventricular function in patients with non-ischaemic cardiomyopathy

**DOI:** 10.1007/s12471-015-0753-y

**Published:** 2015-10-07

**Authors:** M. Martínez-Sellés, E. Pérez-David, R. Yotti, J. Jiménez-Borreguero, G. Loughlin, L. Gallego, A. Ayesta, M.J. Olivera, J. Bermejo, F. Fernández-Avilés

**Affiliations:** 1 Cardiology Department, Servicio de Cardiología, Hospital General Universitario Gregorio Marañón, and Instituto de Investigación Sanitaria Gregorio Marañón, Universidad Complutense de Madrid, and Universidad Europea de Madrid, Calle Dr. Esquerdo 46, 28007 Madrid, Spain; 2 Cardiology Department, Hospital General Universitario Gregorio Marañón, and Instituto de Investigación Sanitaria Gregorio Marañón, Madrid, Spain; 3 Radiology and Cardiology Departments, Hospital Universitario de la Princesa, Madrid, Spain

**Keywords:** Sex, Gender, Right ventricle, Dilated cardiomyopathy, Heart failure

## Abstract

**Aim:**

To evaluate sex-related differences in right ventricular (RV) function, assessed with cardiac magnetic resonance imaging, in patients with stable non-ischaemic dilated cardiomyopathy.

**Methods:**

Prospective multicentre study. We included 71 patients (38 men) and 14 healthy volunteers.

**Results:**

Mean age was 60.9 ± 12.2 years. Men presented higher levels of haemoglobin and white blood cell counts than women, and performed better in cardiopulmonary stress testing. A total of 24 patients (12 women) presented severe left ventricular (LV) systolic dysfunction, 32 (13 female) moderate and 15 (8 women) mild LV systolic dysfunction. In the group with severe LV systolic dysfunction, average right ventricular ejection fraction (RVEF) was normal in women (52 ± 4 %), whereas it was reduced in men (39 ± 3 %) *p* = 0.035. Only one woman (8 %) had severe RV systolic dysfunction (RVEF < 35 %) compared with 6 men (50 %) *p* < 0.001. In patients with moderate and mild LV dysfunction , the mean RVEF was normal in both men and women. In the 14 healthy volunteers, the lowest value of RVEF was 48 % and mean RVEF was normal in women (56 ± 2 %) and in men (51 ±  1 %), *p* = 0.08.

**Conclusions:**

In patients with dilated cardiomyopathy, RV systolic dysfunction is found mainly in male patients with severe LV systolic dysfunction.

Women with systolic heart failure have a better prognosis than men [1], particularly in the case of non-ischaemic dilated cardiomyopathy [1, 2], but the causes of this difference remain unknown [3]. Right ventricular (RV) dysfunction has been associated with impaired exercise capacity and higher mortality in patients with systolic heart failure [3–11], and gender-related differences in RV function in healthy subjects have been suggested [12–14], with higher values of right ventricular ejection fraction (RVEF) in females compared with males [5, 12, 14] in spite of a similar left ventricular ejection fraction (LVEF) [14]. Also, it has been previously reported that women with systolic heart failure less often present with RV dysfunction than men [15, 16]. The superior RV function observed in females could explain, at least in part, why women with non-ischaemic dilated cardiomyopathy have a better prognosis than men.

In spite of the importance of the right ventricle, knowledge regarding this chamber is relatively scarce [17]. This is due, in part, to the complex geometry of the right ventricle, which renders precise measurement of RVEF dimensions and function technically challenging. The existence of RV diastolic dysfunction has been demonstrated in patients with dilated cardiomyopathy and may have a potential role in patient outcomes [18]. Cardiac magnetic resonance imaging (MRI) can accurately measure RV volumes and function and has become the gold standard for the evaluation of the right ventricle, and MRI-derived RV volumes correlate strongly with prognosis in patients with systolic heart failure [19].

Our main aim was to evaluate sex-related differences in RV function, measured with cardiac MRI, in patients with dilated cardiomyopathy and severe left ventricular (LV) systolic dysfunction and no relevant comorbidity. We also assessed sex-related differences in RV function in patients with dilated cardiomyopathy without severe LV systolic dysfunction. Finally MRI was also performed in healthy volunteers, in order to evaluate gender-related RV differences in subjects with no previous cardiac disease.

## Methods

We designed a prospective study, performed in two university hospitals in Madrid, Spain.

## Patients

We enrolled patients with chronic heart failure in whom significant coronary artery disease had been excluded by coronary angiography, who had been clinically stable for at least a month prior to inclusion, and who presented LV systolic dysfunction with LVEF < 30 % measured with echocardiography in the previous 4 months.

Exclusion criteria included all relevant comorbidities: diabetes, clinical criteria for chronic obstructive pulmonary disease or previous spirometry with significantly impaired pulmonary function, history of thromboembolic disease, creatinine > 2 mg/dL, and any systemic disease with a life expectancy < 2 years; hospitalisation for heart failure in the last month; patients with an implanted device that contraindicated MRI (pacemaker, defibrillator, or other); women of childbearing age in whom pregnancy could not be excluded; patients with claustrophobia; patients with significant valvular stenosis or severe aortic regurgitation; patients with congenital heart disease and those who had undergone previous heart transplant.

The study complies with the Declaration of Helsinki and was approved by the Institutional Review Board of *Hospital Universitario Gregorio Marañón*, Madrid, Spain. All subjects provided written informed consent prior to inclusion.

### Clinical evaluation, ECG, non-imaging tests, and treatment

A thorough clinical evaluation was performed in all patients. Twelve-lead ECG was also performed. Blood tests included a complete blood count, chemistry and natriuretic peptide assessment. Cardiopulmonary stress testing (including measurement of respiratory gas exchange) and spirometry were recommended in all patients unless there was a reason not to do so (a number of patients were unable to perform a stress test). A normal spirometry was defined as FVC and FEV1 greater than 80 % of predicted values as well as FEV1/FVC greater than 70 % of the predicted value. Each patient’s drug regime was recorded.

### LVEF and RVEF groups

We divided patients with dilated cardiomyopathy into three different groups according to LVEF measured with MRI: severe (< 25 %), moderate (25–34 %), and mild (35–50 %) LV systolic dysfunction. Regarding RV function we considered three groups: severe RV systolic dysfunction (RVEF < 35 %), moderate RV systolic dysfunction (RVEF 35–44 %), and normal RV systolic function (RVEF > 44 %).

### Healthy volunteers

MRI was performed in 14 healthy volunteers with no previous history of heart disease.

### Imaging

Patients underwent contrast-enhanced MRI as described in the Appendix.

### Statistical analysis

Quantitative variables are reported as mean ± SD, while qualitative variables are reported as numbers and percentages. Continuous variables were compared using Student’s t-test, while categorical variables were compared using the chi-square test, or Fisher’s exact test where appropriate. All statistical analyses were performed using SPSS software (V16, SPSS, Chicago, Ill, USA). All p values are two-tailed.

## Results

### Baseline characteristics and treatment

We enrolled 71 patients, 38 men (53.5 %) and 33 women. Mean age was 60.9 ± 12.2 years. Two patients presented atrial fibrillation (2.8 %), the rest were in sinus rhythm. Baseline clinical characteristics were similar in males and females with the only exception being a higher prevalence of tobacco and alcohol consumption in males (Table 1). Laboratory and functional test results revealed that men presented higher levels of haemoglobin as well as higher white blood cell counts than women, and performed better in cardiopulmonary stress testing (Table 1). Treatment was similar in men and women (Table 2), three patients were not receiving beta blockers due to bradycardia (2 patients) and hypothyroidism, the rest were receiving bisoprolol (3 patients) and carvedilol (65 patients, mean daily dose 32.5 ± 18.8 mg). Ivabradine was not used in any patients. Same-day echocardiography showed severe mitral regurgitation in 6 patients (10.0 %) and moderate mitral regurgitation in 23 patients (38.3 %). Moderate aortic regurgitation was present in 4 patients (6.7 %) and moderate tricuspid regurgitation in 3 (5.0 %). There were no relevant differences regarding valve regurgitation between men and women (data not shown).

**Table 1:** Clinical characteristics and diagnostic test results in males and females.

	Men (*n* = 38)	Women (*n* = 33)	*P* value
**Variables (mean ± SD)**			
Age (years)	60.3 ± 13.2	61.5 ± 11.4	0.70
Weight (Kg)	76.2 ± 10.6	70.6 ± 22.9	0.18
Height (cm)	170.3 ± 7.9	155.0 ± 13.6	< 0.001
Body mass index	26.2 ± 2.6	27.6 ± 5.6	0.17
Systolic BP (mmHg)	121 ± 23	121 ± 21	0.99
Heart rate (bpm)	74 ± 14	72 ± 10	0.62
Years from DCM diagnosis	2.7 ± 3.5	2.1 ± 2.7	0.44
**Variables** *N* **(%)**			
Tobacco	26 (68.4)	11 (33.3)	0.004
Alcohol	18 (47.3)	1 (3.0)	< 0.001
Functional class I/II/III (%)	31.6/57.9/10.5	24.2/57.6/18.2	0.33
Previous HF admissions	32 (84.2)	24 (72.7)	0.25
Hypertension	14 (36.8)	13 (39.4)	0.83
Hyperlipaemia	13 (34.2)	14 (42.4)	0.49
**ECG**			
QRS duration (mean ± SD)	120 ± 34	122 ± 29	0.71
LBBB N (%)	15 (39.5)	17 (51.5)	0.32
**Blood tests (mean ± SD)**			
Hemoglobin g/dL	14.3 ± 1.4	13.3 ± 1.2	0.002
Leukocyte count/µL	8058 ± 1862	6761 ± 1996	0.008
BNP pg/mL	196 ± 223	331 ± 373	0.12
NT-proBNP pg/mL	1808 ± 2536	1651 ± 2194	0.84
**Stress test (mean ± SD)**			
METs	6.8 ± 2.0	5.1 ± 1.6	0.002
Peak O2 consumption ml/kg/min	23.9 ± 6.3	18.4 ± 4.7	0.001
**Spirometry**			
FEV1/FVC (Mean ± SD)	73.8 ± 8.4	76.4 ± 6.6	0.19
Normal spirometry (%)	22 (62.9)	10 (35.7)	0.03
RVEF if normal spirometry	52.9 ± 13.1	52.7 ± 10.9	0.97
RVEF if abnormal spirometry	48.1 ± 11.5	59.8 ± 11.8	0.01

*BP* blood pressure, *DCM* dilated cardiomyopathy, *HF* heart failure, *LBBB* left bundle branch block, *BNP* B-type natriuretic peptide, *NT-proBNP* amino-terminal pro BNP, *METs* metabolic equivalents, *FEV1* forced expiratory volume in 1 s, *FVC* forced vital capacity.

**Table 2:** Medical treatment in males and females.

	Men (*n* = 38)	Women (*n* = 33)	*P* value
Variable *N* (%)			
Beta blockers	36 (94.7)	32 (97.0)	0.65
ACEI	33 (86.8)	28 (84.2)	0.81
ARB	7 (18.4)	8 (24.2)	0.56
Digoxin	5 (13.2)	2 (6.1)	0.32
Aldosterone antagonists	18 (47.4)	12 (36.4)	0.93
Diuretics	14 (36.8)	12 (36.4)	0.97
Statins	12 (31.6)	8 (24.2)	0.50

*ACEI* angiotensin-converting-enzyme inhibitors, *ARB* angiotensin II receptor blocker.

### RVEF according to LVEF

**Fig. 1:**
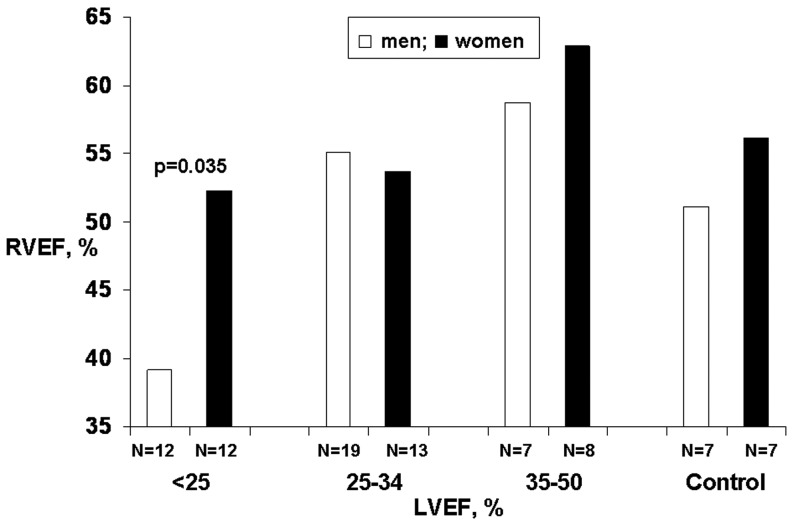
Mean right ventricular ejection fraction (RVEF) according to left ventricular ejection fraction (LVEF) group in men and women. Data from 71 adults with stable dilated cardiomyopathy and 14 healthy volunteers.

**Fig. 2:**
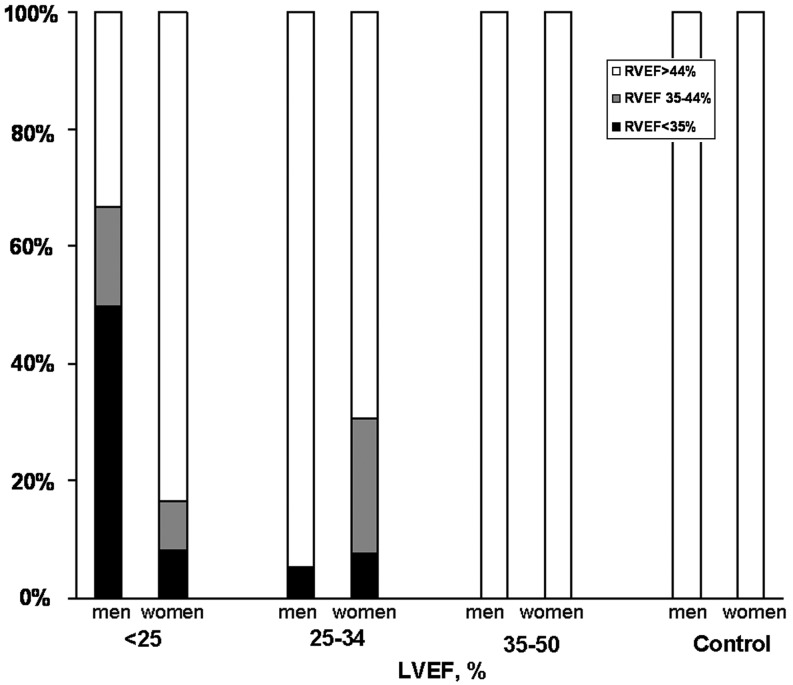
Right ventricular ejection fraction (RVEF) group according to left ventricular ejection fraction (LVEF) group in men and women. Data from 71 adults with stable dilated cardiomyopathy and 14 healthy volunteers.

A total of 24 patients (12 women) presented severe, 32 moderate (13 women) and 15 mild (8 women) LV systolic dysfunction. Amongst patients with severe LV systolic dysfunction, women presented a higher mean RVEF (52 ± 4 % *vs*. 39 ± 4 %, *p* = 0.035) (Fig. 1); moreover, only one woman (8.3 %) had severe right systolic dysfunction compared with 6 men (50.0 %), *p* < 0.001 (Fig. 2). In patients with moderate and mild LV dysfunction mean RVEF was normal in both men and women (Fig. 1).

We enrolled 14 healthy volunteers (mean age 45.2 ±  7.3 years, 7 men). The lowest value of RVEF was 48 % and mean RVEF was normal in women (56.1 ± 2.3) and in men (51.1 ± 1.1), *p* = 0.08.

### Other MRI parameters

Late enhancement was assessed after injection of gadodiamide in 65 patients (34 men). Despite the absence of significant coronary artery disease in coronary angiography, subendocardial late enhancement was present in 5 men (14.7 %) and no women, *p* < 0.01. No significant differences were found in average pulmonary artery flow velocity between female and male patients, or between healthy men and women (Table 3). However, average pulmonary artery flow velocity was significantly lower in patients compared with healthy volunteers (10.3 ± 3.2 versus 15.3 ± 3.4, *p* < 0.001). A significant correlation existed between pulmonary artery flow velocity and RVEF (*r* = 0.35 *p* = 0.003).

**Table 3:** Magnetic resonance imaging (MRI) results in males and females.

**Patients**	**Men (** ***n*** **= 38)**	**Women (** ***n*** **= 33)**	***P*** **value**
**Variable (mean ± SD)**			
LV mass (g)	147.8 ± 48.1	121.5 ± 44.0	0.02
LVEF (%)	28.4 ± 10.1	28.9 ± 10.3	0.84
RVEF (%)	50.7 ± 12.7	55.4 ± 13.5	0.14
LVEDV (mL)	312.6± 107.5	267.9 ± 100.1	0.08
LVESV (mL)	231.6 ± 108.5	198.8 ± 97.6	0.19
RVEDV (mL)	157.9 ± 47.5	132.5 ± 46.7	0.03
RVESV (mL)	80.9 ± 43.3	65.2 ± 44.0	0.13
iLVEDV (mL/m^2^)	123.1 ± 58.8	115.8 ± 56.0	0.49
iLVESV (mL/m^2^)	231.6 ± 108.5	156.7 ± 56.6	0.60
iRVEDV (mL/m^2^)	83.4 ± 25.5	77.4 ± 25.6	0.32
iRVESV (mL/m^2^)	42.8 ± 23.4	38.0 ± 25.1	0.40
Late enhancement (%) no/DCM typical/DCM atypical/ischaemic	50.0/26.5/8.8/14.7	64.5/22.6/12.9/0	0.03
Average PA flow velocity (cm/s)	10.5 ± 3.1	10.0 ± 3.4	0.57
MRI-estimated PVR	4.7 ± 2.0	4.6 ± 2.4	0.8
**Volunteers**	**Men (** ***n*** **= 7)**	**Women (** ***n*** **= 7)**	***P*** **value**
**Variable (mean ± SD)**			
LV mass	85.6 ± 27.1	64.9 ± 11.0	0.08
LVEF	62.3 ± 5.3	60.6 ± 5.2	0.55
RVEF	51.1 ± 3.1	56.1 ± 6.0	0.07
Average PA flow velocity (cm/s)	15.8 ± 2.9	14.8 ± 4.3	0.6
MRI-estimated PVR	2.5 ± 0.8	1.7 ± 2.4	0.2

*LV* left ventricular, *LVEF* left ventricular ejection fraction, *RVEF* right ventricular ejection fraction, *DCM* dilated cardiomyopathy, *LVEDV* left ventricular end-diastolic volume, *PA* pulmonary artery, *LVESV* left ventricular end-systolic volume, *RVEDV* right ventricular end-diastolic volume, *RVESV* right ventricular end-systolic volume, *i* index, *PVR* pulmonary vascular resistance.

### Spirometry and stress test

Spirometry was performed in 64 patients, revealing normal flow-volume curves in 32 (45.1 %), reduced mid expiratory flow in 14 (19.7 %), moderate obstruction in 7 (10.9 %), mild obstruction in 6 (8.5 %), and a mild restrictive pattern in 5 patients (7 %). The prevalence of a normal spirometry was similar in patients with or without previous tobacco use (47.1 % vs 55.2 %, *p* = 0.53). A normal spirometry was more often found in men than in women (Table 1). In patients with a normal spirometry, RVEF was similar in men and women; however in patients with an abnormal spirometry result RVEF was lower in men. Also, compared with patients with abnormal spirometry results, patients with normal flow-volume curves performed better during stress testing, 5.1 ±  1.9 METs vs 6.8 ± 1.9 METs, *p* = 0.005 and had a higher peak oxygen consumption 18.5 ± 5.7 ml/kg vs 24.3 ± 5.7 ml/kg, *p* = 0.01. Patients with and without RV dysfunction had similar results in stress tests, 5.8 ± 2.3 METs vs 5.9 ± 1.9 METs, *p* = 0.90; peak oxygen consumption 21.2 ± 6.0 ml/kg vs 20.8 ± 6.7 ml/kg, *p* = 0.84.

## Discussion

In our cohort of dilated cardiomyopathy patients, RV dysfunction was found mainly in male patients with severe left systolic dysfunction, suggesting a degree of protection from RV dysfunction amongst women. Our data confirm that RV dysfunction is unusual in patients with dilated cardiomyopathy; also, as expected, RV systolic dysfunction was found almost exclusively in patients with a very low LVEF [16]. However, the main finding of our study is that, in this subgroup of patients with severely depressed LV systolic function, most men also presented RV systolic dysfunction, whereas this finding was unusual in women.

Multiple studies in heart failure patients have consistently found a survival benefit in females, which is more pronounced in non-ischaemic heart failure [17]. However, the underlying causes for this improved prognosis remain unknown [3]. The largest study questioning the role of the right ventricle in chronic systolic heart failure was performed by Meyer et al. [5], using the Beta-Blocker Evaluation of Survival Trial (BEST) dataset, with RVEF measured with gated-equilibrium radionuclide ventriculography. These authors found that, compared with patients with normal RVEF, there were fewer women amongst those with a lower RVEF. We have also previously suggested, in a study performed with echocardiography, that the prevalence of RV dysfunction in patients with systolic heart failure is lower in women than in men [15]. Low RVEF is a strong predictor of poor outcome in patients with systolic heart failure [4]. A recent meta-analysis has confirmed the significant association between RV systolic dysfunction and overall mortality [20]. Thus, it is conceivable that the better performance of the right ventricle encountered in women with chronic systolic heart failure could, at least in part, explain their more favourable prognosis [1].

Assessment of the right ventricle with echocardiography is problematic [21], as it depends on both an adequate acoustic window and, due to the crescent shape of the right ventricle, on making certain geometric assumptions for the calculation of ventricular volumes. Identifying an accurate and reliable echocardiographic parameter for the functional assessment of the right ventricle still remains a challenge [21] and, although gated-equilibrium radionuclide ventriculography is an alternative, it also entails significant limitations, most importantly a poor spatial resolution [22]. We designed our study with the intent of confirming the superior performance of the female right ventricle in chronic systolic heart failure providing the most comprehensive and accurate assessment of RV dimensions and function available. In this sense, cardiac MRI has excellent spatial resolution, can accurately measure volumes and RVEF without relying on geometric assumptions and is, in short, the gold standard for the non-invasive evaluation of the right ventricle [19]. Also, to avoid the presence of confounding variables, we selected patients with chronic dilated cardiomyopathy, optimal medical treatment, and no comorbidity.

Although biventricular involvement can be occasionally found in non-ischaemic dilated cardiomyopathy, by itself, intrinsic myocardial damage is usually not sufficient to lead to RV failure, and an additional stressor is often necessary to provoke it [19], mainly in the form of increased pulmonary artery pressures, itself a consequence of LV dysfunction. In fact, an increase in RV afterload through the development of pulmonary arterial hypertension secondary to chronic pulmonary venous hypertension has long been considered the main underlying mechanism of RV failure [5] and pulmonary wedge pressure is the strongest predictor of RV dysfunction [23]. Phase contrast MR imaging is useful for non-invasive detection of pulmonary arterial hypertension. In a recent study published by Sanz et al., average pulmonary artery flow velocity showed good correlation with pulmonary pressures [24]. However, in our study, lower pulmonary pressures do not seem to explain the better performance of the female right ventricle in patients with very low LVEF. In fact, although average pulmonary artery flow velocity was significantly higher in patients with dilated cardiomyopathy than in healthy volunteers, no significant differences existed in pulmonary artery flow velocity between men and women. Nonetheless, patients with previous lung disease were not included in our study and a normal spirometry was more frequently found in men than in women. Interestingly, sex-related differences regarding RVEF were seen in patients with a pathological result in spirometry. Areas of ischaemic late enhancement were present in 5 men (only 2 of them had LVEF < 25 %) and no women; this small number is not enough to explain the differences regarding RVEF.

The underlying reasons for the superior performance of the female right ventricle in patients with dilated cardiomyopathy is currently unknown. Oestradiol levels have been associated with better RV systolic function [25]. Another hormone, relaxin, is secreted by the ovaries and the placenta, with increased levels during pregnancy that mediate some of the haemodynamic changes associated with gravidness. Serelaxin, recombinant human relaxin-2, has recently been found to improve the prognosis in patients with heart failure [26]. Finally, the presence of XY chromosome positive cardiomyocytes in the hearts of women who have had male offspring has been reported [27]. Although the implications of this important finding are still unclear, it cannot be ruled out that the heart undergoes some degree of rejuvenation during pregnancy, which would provide an advantage in the event that the subject were to later develop dilated cardiomyopathy.

Although not gender-related, an additional interesting finding was the correlation between spirometry results with functional capacity and peak oxygen consumption, suggesting a possible role of spirometry in risk stratification of these patients. Of note, lung function variables obtained by spirometry are frequently impaired in patients with heart failure and correlate with all-cause mortality [28] and a restrictive pattern in spirometry predicts poor survival in chronic heart failure [29].

Several limitations of our study need to be considered. The present work is based in a relatively low number of highly selected patients with chronic dilated cardiomyopathy and no comorbidity, thereby potentially limiting the application of our findings to larger non-selected populations as well as the power to detect the influence of certain factors such as the previous number of pregnancies. Also, the low number of healthy volunteers clearly precludes establishing definitive conclusions. Indeed, we were unable to confirm certain previously described sex-related differences [13, 14, 30], specifically a higher average RVEF in healthy females compared with males [14, 30]. Moreover, the concept of a normal right ventricle is unclear, as RV wall motion abnormalities have been recently described in healthy subjects [31]. Finally, left and right ventricular systolic volumes are correlated and both are used to calculate ejection fraction. However, to the best of our knowledge, no study to date has examined the association of gender with RV function in optimal conditions (MRI, a population with ‘pure’ dilated cardiomyopathy and no comorbidity).

## Conclusions

In chronic stable dilated cardiomyopathy patients, RV systolic dysfunction is found mainly in male patients with severe LV systolic dysfunction; on the other hand, females with severe LV systolic dysfunction present a normal mean RVEF.

### Funding

This work was supported by the grant ‘[*PI070837*]’ and the Red de Investigación Cardiovascular ‘[RD12/0042]’ from the *Instituto de Salud Carlos III, Madrid, Spain*.

### Conflict of Interests

None declared.

## Appendix

Patients underwent contrast-enhanced MRI (ce-MRI) with a 1.5-T scanner (Philips Intera®, Best, the Netherlands) equipped with a Nova gradient system (33 mT/m; 160 mT/m/ms). A five-element dedicated cardiac coil was used. All images were obtained with ECG gating and breath-holding. The MRI study consisted of cine steady-state free-precession imaging of left and right ventricular function (SENSE × 2, repetition time, 2.4 ms; echo time 1.2 ms, FOV 360 mm (up to 390 mm in obese patients to avoid fold-over), 30 phases per cycle, 8-mm slice thickness without gap) in axial views (10 to 15 contiguous slices from pulmonary artery bifurcation to inferior vena cava), short axis views (10 to 14 contiguous slices from mitral annulus to LV apex, achieving full coverage of the left ventricle) and 4-chamber, 2-chamber and 3-chamber views. Phase-contrast MR images perpendicular to the main pulmonary artery and aorta were acquired with a segmented fast gradient echo MR sequence, with velocity encoding perpendicular to the imaging plane and a predefined upper velocity limit of 100 cm/sec for the main pulmonary artery and 150 cm/s for the aorta. If aliasing was noted, the velocity was progressively raised in 30-cm/sec steps until it disappeared. Late enhancement imaging (3D inversion recovery turbo gradient echo sequence, prepulse delay optimised for maximal myocardial signal suppression; repetition time 3.4 ms, echo time 1.3 ms, spatial resolution 1.5 × 1.8 mm, 5-mm slice thickness, inversion time 200-300 ms) was performed in patients with dilated cardiomyopathy 10 to 15 minutes after a total injection of 0.2 mmol/kg gadodiamide (Omniscanâ, GE Healthcare). Late enhancement images were obtained in the same short and long axis views defined for cine imaging. An echocardiogram was performed in the same day in 60 patients (84.5%).

## Ce-MRI data analysis

MRI analysis was performed by an investigator (EPD) with 10 years of experience in MRI, blinded to patient clinical data. Cine, phase contrast, and late enhancement images were reviewed and analysed off-line in DICOM format with specialised postprocessing software (QMass® MR 7.0, QFlow 5.0 MEDIS, the Netherlands). Left and right ventricular end-diastolic and end-systolic volumes were assessed based on the Simpson’s rule and LVEF and RVEF were obtained. LV mass was calculated by substracting the endocardial volume from the epicardial volume at end diastole and then multiplying by the tissue density (1.05 g/ml). Axial views were used for RV and short-axis views for LV measurements. The following parameters were calculated from phase contrast images: peak velocity, average velocity, minimum and maximum areas of aorta and pulmonary artery. Pulmonary artery pulsatility (relative change in lumen area during the cardiac cycle) was calculated as: maxA – minA/minA × 100. Late gadolinium-enhanced images were visually analysed to identify intramyocardial areas of enhancement. Four patterns were defined: a) No enhancement; b) Subendocardial or transmural enhancement; c) Typical non-ischaemic dilated cardiomyopathy, i.e. longitudinal or patchy midwall enhancement, not corresponding to the territory of a coronary artery and not subendocardial; d) Other atypical patterns not involving subendocardium. An area of enhancement was only deemed to be present if it was seen in a short axis view and a cross-cut long axis image.
